# Unequal ends: A systematic review comparing place of death in rural, coastal and urban areas for older people with dementia to cancer

**DOI:** 10.1177/02692163261426428

**Published:** 2026-03-18

**Authors:** India Tunnard-Johnson, Rachel L. Chambers, Peter May, Frank Jackson-Hill, Irene J. Higginson

**Affiliations:** 1 Department of Palliative Care, Policy and Rehabilitation, Cicely Saunders Institute, King’s College London, UK; 2 School of Medicine, Trinity College Dublin, Ireland

**Keywords:** dementia, neoplasms, rural population, urban population, sea coast, systematic review

## Abstract

**Background:**

Despite the growing older population with increased chronic conditions in rural and coastal areas, it is currently unclear how location impacts where people die.

**Aim:**

To compare where people with dementia and people with cancer die across rural, coastal and urban areas, and examine associated factors.

**Design:**

A systematic review reported in accordance with PRISMA guidelines.

**Data sources:**

MEDLINE, PsycINFO, CINAHL, Embase and ASSIA databases were searched 2005–November 2024.

**Results:**

Twenty-nine studies were included. Fifteen studies compared rural and urban populations, eleven urban only, two coastal urban and one rural. No studies considered the coastal context when examining place of death. 6,988,050 cancer and 3,517,909 dementia decedents were included across seventeen cancer studies, nine dementia, and three both. All studies were rated high quality. Rural, dementia decedents were more likely to die in residential care than urban residents, who were more likely to die in hospital. Large studies of cancer populations found trends towards home deaths for rural residents and hospital for urban, but this was inconsistent across countries. Thirteen studies reported factors associated with place of death for people with cancer, compared to four dementia.

**Discussion:**

Hospital deaths appear more common in urban areas, with more home/care home deaths in rural areas. Coastal patterns are poorly described. Better understanding how these locations are associated with place of death, especially for people with dementia, is needed to guide more equitable, efficient service planning. Further work on associated factors will support a nuanced understanding of drivers of place of death.

**What is already know about the topic?**

- Understanding place of death is vital for service planning and delivery.
- People with dementia often experience less access to palliative care and other supportive services at the end of life, as compared to people with cancer.
- The rural and coastal older populations are growing and predicted to grow further in the coming years alongside increases in chronic conditions, such as dementia and cancer.

**What the paper adds?**

- This review found that hospital deaths are more likely for urban residents and home deaths are more likely for all rural residents.
- A disproportionate amount of palliative and end of life care research focuses on urban populations, despite many older people living outside of urban areas. When rural populations are considered, this is often in comparison to urban populations. Coastal populations are little considered.
- Factors associated with place of death in rural and coastal areas and for people with dementia remain underexplored.

**Implications for practice, theory or policy**

- Alongside urban and rural classifications, coastal settings should be explicitly recorded and analysed to better understand and plan for variations in place of death.
- The refinement of the conceptual model could inform the allocation of resource to support service expansion and delivery in rural and coastal areas, but further research is required.
- It is recommended that future research focuses on where rural and coastal populations are likely to die and the factors that are associated with place of death in these areas, with a particular focus on service-level factors and preferences.

## Introduction

Understanding where people die is vital to support the planning and delivery of health and social care services.^1^ However, where people are dying is changing.^1–5^ There are a multitude of reasons why, including expansion in care home bed availability, changes in governmental policy and ‘shifts’ in causes of death,^3,4^ among others. It is not only the setting (i.e. hospital and home) in which people are dying that is changing^1^ but the geographical location in which people are dying is changing too. Specifically, the number of deaths in rural and coastal areas is rising and is largely driven by increasingly older populations.^6–9^ As people age, they tend to move out of urban areas into rural and coastal areas in retirement, while younger people tend to leave for education and employment opportunities in large cities. This results in fewer younger residents to support the older population and to fill roles in the health and social care workforce.^10,11^ Adding to the population challenges, rural and coastal populations often experience issues accessing healthcare due to inadequate travel and transport infrastructures^12,13^ to urban health service hubs. This is a particular disadvantage for the older population in coastal areas who experience an age- and sex-adjusted excess of chronic conditions, such as dementia and cancer, compared to their inland counterparts.^11^ This is going to become an increasingly pressing issue for these communities as the number of people living with dementia is projected to more than double in 20 years^14^ and, alongside cancer, see the largest increases in deaths requiring palliative care support.^15^

The conditions people live with in their final years play a large contributing role in where they die, with people with cancer more likely to die at home^16,17^ and people with dementia more likely to die in a care home or hospital.^3,17^ Place of death is also often used as a marker of quality of care at the end of life^18,19^ with home deaths seen as indicative of high-quality care that is aligned with patient and family preferences.^20^ Home deaths also tend to be more likely for people with cancer due to improved access to palliative care services, and the opportunity to actively discuss their preferences with family.^16,21–24^ Conversely, people with dementia often experience poorer access to specialist palliative care services^25,26^ that might enable them to stay at home to die^27^ and potentially less able to communicate wishes at the end of life.^28^ Instead, many people with dementia die in a hospital,^3,29^ which is indicative of aggressive care at the end of life^18^ and the preference of few.^30^ As people with dementia also tend to be older than people with cancer, it may also mean that they are more likely to live in a rural area with reduced access to specialist palliative care, and other, services.

Geographical locations, such as rural, coastal and urban areas, have received little research attention thus far. Where reviews have examined health outcomes in varying locations, they more often focus on the accessibility and availability of care services in these areas,^13,31–33^ and place of death is overlooked. Further, where there is existent examination of the evidence, it often focuses on rural and urban locations with coastal areas missing from the evidence base, despite the identified increased presence of chronic conditions in coastal areas.^11^ Where place of death is considered, the focus of the review is often condition specific with rural/urban location considered as a factor associated with place of death.^22,34^ Further, these reviews group associated factors around: the individual, such as age and sex, the illness, such as severity and function, the environmental, such as household set up and access to healthcare services, and services such as healthcare availability, but do not consider how these factors may vary by urban, rural and coastal locations.

Therefore, the primary aim of this systematic review was to examine the international evidence base on where older people with dementia and older people with cancer die in rural, coastal and urban areas. In addition, we aimed to identify any factors associated with place of death by location and condition.

## Methods

The reporting of this systematic review followed Preferred Reporting Items for Systematic Reviews and Meta-Analyses (PRISMA) checklist (Supplemental Appendix 1). This review was not registered but the review protocol can be accessed via the corresponding author.

### Search strategy

Five databases, MEDLINE, PsycINFO, CINAHL, Embase and ASSIA, were searched for papers published in English from January 2005 to 21st November 2024. The search strategy was developed with support from an information specialist and included terms related to place of death, condition and geographical location (Supplemental Appendix 2). Reference chaining and citation tracking were undertaken to complement the search strategy.

### Eligibility criteria

Criteria for assessing the relevance of selected papers was outlined using the PICO-ST (Population, Intervention, Comparator, Outcome – Study design, Time) framework (Table 1).^35^

**Table 1.** Eligibility criteria according to PICO-ST framework.^35^

PICO-ST criteria	Inclusion criteria	Exclusion criteria
Participants	All older adult decedents with any type of dementia or cancer (populations must >80% with dementia or cancer and over the age of 65 years or results stratified by age or, where age isn’t provided, type of cancer more typical in older age)	Accidental deaths Population <65 years old (<80% are over 65 years) Deaths of or with conditions other than dementia or cancer (<80% do not have cancer/dementia)
	All studies where place of death is stratified by named geographical city, town or village, or is defined as ‘rural’, ‘coastal’ or ‘urban’	Geographical location (i.e. name of city/town/village or rural/coastal/urban categorisation) of death is not reported
Interventions	Any	-
Comparators	Any	-
Outcomes	Place of death	Any other outcome including survival, mortality Any studies examining the effects the COVID-19 pandemic
Study design	Any design reporting original quantitative data	Opinion pieces, conference abstracts, presentations, editorials, case reports, case-series, reviews, protocols, and qualitative designs.
Time	Studies conducted in the last 20 years (where longitudinal data is collected, only data from 2004 onwards is extracted)	Studies published or include data collected prior to 2004

Papers were included that detailed the rural, coastal and/or urban location and setting of death for people who died of any type of dementia or cancer, aged 65 years or older. Factors associated with place of death were extracted from studies where rural, coastal and/or urban location could be determined.

### Study selection

Identified papers were managed in EndNote 20 reference management system. One researcher (ITJ) reviewed the title and abstracts of all identified papers for inclusion. The same researcher reviewed all full text papers for inclusion, with 20% double checked by a blind researcher (RLC). Any disagreements in included papers were resolved through discussion.

### Quality appraisal

Quality appraisal was conducted using the QualSyst tool^36^ for all included papers. QualSyst is a standardised 14-item tool developed for the assessment of quality of published quantitative studies. Quality appraisal was used in the interpretation of findings and therefore, no paper was excluded based on the quality assessment. Papers were graded as high (⩾0.8), medium (⩾0.6–0.79) and low (<0.6).^34,37^ All papers were appraised by two researchers (ITJ and RLC), with disagreement or divergence in scores discussed and remedied.

### Data extraction and synthesis

Data extraction was completed using a bespoke Excel template. The template was informed by the review aims and included lead author, date of publication, country of study, paper aims, design, condition of sample, setting (i.e. hospital, hospice) of death, findings related to rural, coastal and/or urban location and associated factors. Data was extracted by one researcher (ITJ) with 100% checked independently by another researcher (RLC).

The synthesis was guided by Gomes and Higginson’s model of factors associated with place of death.^22^ Factors associated with place of death by rural, coastal and/or urban location and condition were categorised by individual (i.e. demographics and preferences), illness (i.e. length or severity of illness and function), environmental (i.e. social support and heath care use) and service factors (i.e. service availability).

The heterogeneity of included studies resulted in a meta-analysis being inappropriate. Therefore a narrative synthesis was undertaken using Popay’s guidance.^38^

## Results

**Figure 1.**
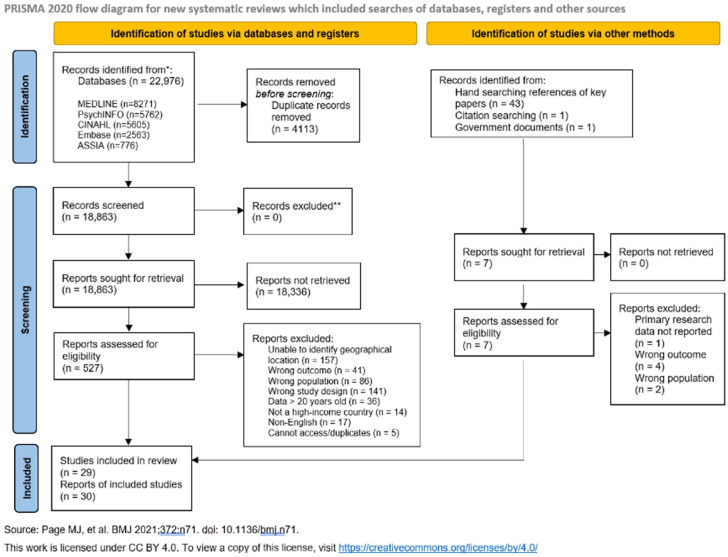
PRISMA flow diagram.

The searches identified 18,863 papers for review, and an additional 45 papers were identified from other methods (Figure 1). Thirty papers have been included in this review, detailing 29 individual studies (see Supplemental Appendix 3 for full details). Most papers were excluded as the geographical location was not, or could not be, categorised. Ten studies were conducted in the UK, four in Canada, three in Belgium, three in USA, two in each Japan, Australia, and Sweden and one study from each: Italy, China, South Korea. Sample sizes ranged from 32 to 2,778,592 (Supplemental Appendix 3). One experimental study design was included, but this was not randomised. Most studies were observational cohort studies.

All studies were of high quality and graded 0.8–1. Studies were graded lower due to missed reporting on study design and sample size estimates, and lack of robust outcome measures.

Fourteen studies examined place of death in rural and urban populations^3,39–52^, twelve studies focussed only on urban populations^24,53–63^, two on costal urban populations^64,65^ (both categorised based on named geographical location) and one study focussed only on a rural population.^66^ No studies were identified looking at coastal rural areas and nor was the term ‘coastal’ used as a geographical area for examination by any study.

**Figure 2.**
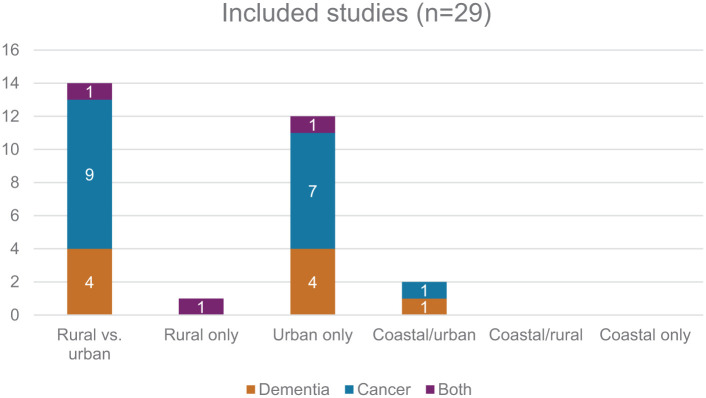
Studies included in the review by condition and geographical location.

A total of 3,517,909 dementia decedents and 6,988,050 cancer decedents were included in the studies. Three studies^52,55,66^ included both dementia and cancer populations, nine focussed on dementia^3,41,48,51,53,58,61,63,65^ and seventeen cancer^24,39,40,42–47,49,50,54,56,57,59,60,62,64^ (Figure 2).

### Definition of geographical locations

Where provided, the definitions of urban and rural locations were heterogeneous. The most common definition of a rural area was a census area with less than 10,000 residents. This definition was used by three Canadian studies^39,48,50^ and one UK study.^3^ One American study defined rural as an area with less than 50,000 residents^41^ and another as less than 2,500.^45^ Most studies used definitions provided by local governing bodies.^3,41,44,45,47,48,50^ No study provided a definition for a coastal area.

### Place of death by geographical location

#### Rural and urban studies

Fourteen studies examined place of death in rural and urban areas with four focussing on people with dementia^3,41,48,51^, nine on people with cancer^39,40,42–47,49,50^ and one included both populations.^52^

Studies of dementia decedents found that hospital deaths were more likely as the area got more urbanised, and that for rural residents, a care home or home death was more likely.^3,41,48,51^ The sample sizes included in these studies ranged from 2,778,592 to 63,375 and all were very high quality.

In the largest two studies of cancer decedents (sample sizes *n* = 5,570,065 and 815,780),^40,49^ an increase in home deaths for rural residents was found (55% in metro vs 85% in non-metro areas). Further, as a place got less urbanised, the chances of dying at home increased (e.g. England: average urbanisation OR 1.2, 95% CI 1.20–1.28, Rural: OR 1.4, 95% CI 1.28–1.55). However, this varied by country. Findings from Canada, USA, France and Sweden report increased home deaths in urban areas.^40,45,47,50^ In China, being on a rural insurance plan, compared to an urban plan, increased the odds of dying in hospital for people with cancer (OR = 7.1, 95% CI 4.15–12.0, *p* < 0.001).^46^

One study that included dementia and cancer decedents^52^ did not provide data on place of death prior to the hospice care intervention, therefore findings are reported under environmental factors.

#### Rural studies

One study examined all deaths in rural Scotland^66^ with a large sample size (*N* = 19,697). A higher proportion of people with dementia died residential care (*n* = 545; 72%), compared to people with cancer (*n* = 3105), who mostly died in acute hospitals (52%), home (28%) and specialist palliative care units (27%).

#### Urban studies

Twelve studies examined place of death in urban areas only. Four included dementia decedents only^53,58,61,63^, seven cancer^24,54,56,57,59,60,62^ and one both.^55^

All studies of dementia decedents took place in the UK. They found that people with dementia living in urban areas of the UK mostly died in a care home (42%–81%) and only a small proportion died at home (11%–27%). The largest study, where preferences were documented, reported the smallest proportion of hospital deaths (12%) and the only study from outside of London reported the highest (47%). The largest two studies including cancer decedents only were also conducted in the UK. They found that 50%–54% died in hospital, 18%–20% died in hospice, and 19%–20% in their own home.^57,59^ In smaller studies of cancer decedents, where participants were receiving palliative care, the majority died at home^60,62^ or in a hospice^56^

One study examined all deaths in Belgium^55^ and found that 74% of people with Alzheimer’s died in residential care, whereas 74% of people with cancer died in a hospital.

#### Coastal urban studies

In Sweden, cancer decedents who received conventional care mostly (63%) died in hospital.^64^ Similarly, approximately half (49%) or people with dementia died in hospital in coastal Australia.^65^

### Factors associated with place of death

**Figure 3.**
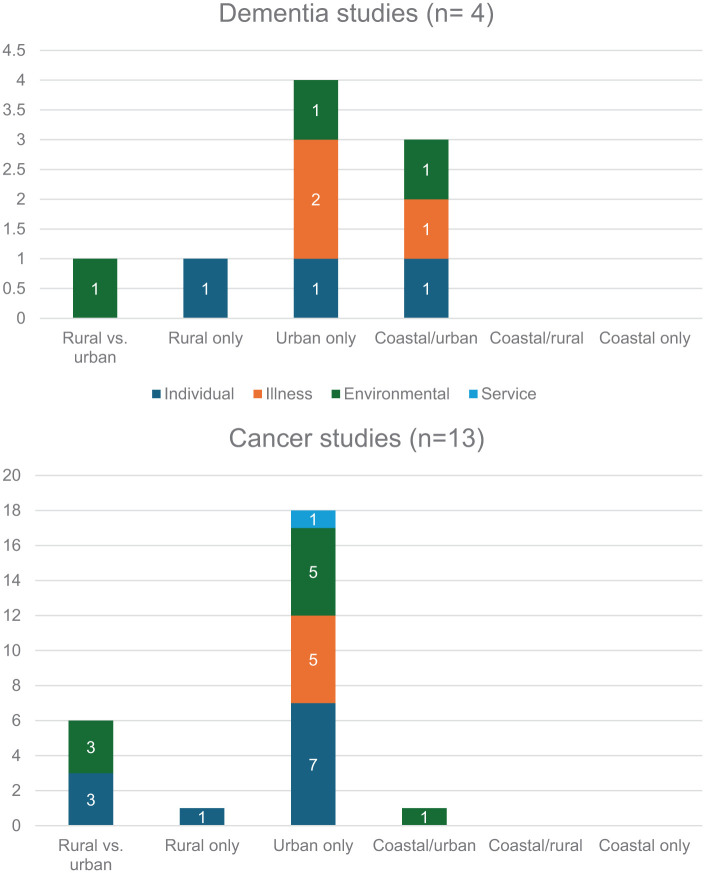
Factors associated with place of death identified within included studies.

**Figure 4.**
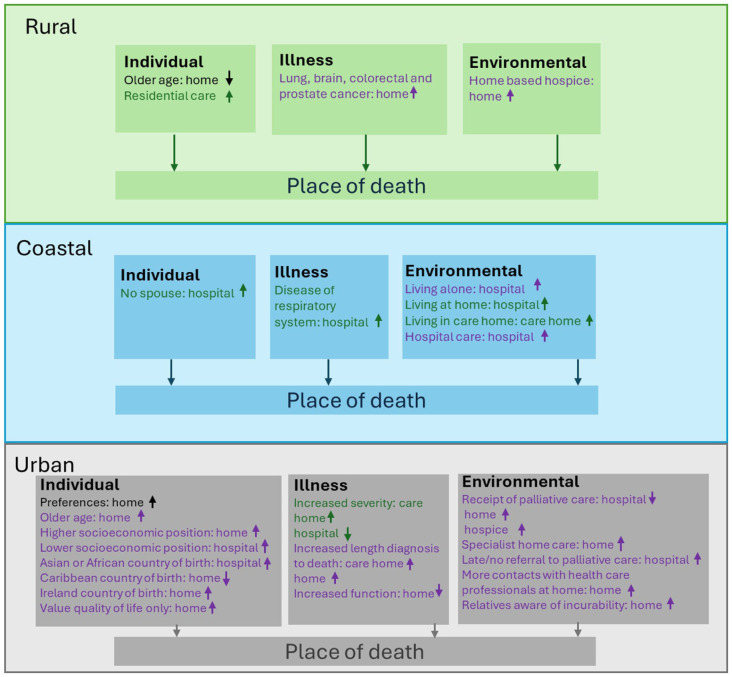
Factors associated with place of death for people with dementia and people with cancer. Factors associated with both dementia and cancer populations; dementia only; cancer only.

Most studies that examined factors associated with place of death were undertaken in urban populations^24,53–57,59,60,62,63^ or in urban populations compared with rural populations.^44–46,50,52^ Only two coastal urban studies^64,65^ and one rural study^66^ examined factors. None of the identified associated factors were examined across rural, coastal and urban areas. Thirteen studies examined factors associated with place of death for people with cancer, only four included studies examined factors associated for people with dementia (Figure 3). For people with dementia, most factors examined were around the illness (*n* = 4), whereas for cancer, they were mostly around the individual (*n* = 11) and environment (*n* = 9) (see Figure 4).

### Individual factors

The most commonly examined factors associated with place of death were around individual factors. Fourteen studies examined individual factors, including age, socioeconomic position, country of birth, marital status and preferences.

#### Rural and urban studies

Both studies examining factors associated with place of death in rural and urban areas only examined socioeconomic factors in cancer populations.^46,50^ At an area level, residents in the highest income urban neighbourhood were 69% more likely to die at home, compared to residents in the lowest income neighbourhood (OR 1.7, 95% CI 1.54–1.85).^50^ Whereas, at an individual level, those with a lower socioeconomic status (indicated by insurance type) in urban areas had increased odds of dying in hospital (OR 28.0, 95% CI 16.8–46.6, *p* < 0.001).^46^

#### Rural studies

In rural Scotland, home deaths were less likely with increasing age (OR 1.0, 95% CI 0.97–0.98).^66^ However, compared to people with cancer, the odds of dying in residential care over a hospital increased with age for people with dementia (OR 20.3, 95% CI 14.51–28.29).

#### Urban studies

The most commonly examined individual factor was age. Large studies of cancer decedents found older age to be associated with home deaths, compared to hospital.^54,62^ Smaller studies found no association.^56,60^ Country of birth also appeared to have some association with place of death for cancer populations. A large UK study^57^ found that compared to people born in the UK, hospital deaths were more likely for those born in Asia (PR 1.1 (1.08–1.15)) and Africa (PR 1.1 (1.07–1.16)). Deaths in own home were significantly less likely for those born in the Caribbean (PR 0.9 (0.85– 0.98)) and more likely for people born in Ireland (PR 1.1 (1.07–1.19)).

For both people with cancer and people with dementia, preference appears to be associated with place of death. Of people with dementia who stated a preference for place of death, 84% were able to achieve it, leading to the majority dying in a care home or at home (51% and 27% respectively).^63^ Whereas, for people with cancer, home deaths were more likely when patients discussed their preferences with family (AOR, 3.4; 1.63–7.04; OR 16.5, 95% CI 3.30–82.73).^24,60^

#### Coastal urban studies

In dementia populations, people without a listed spouse were more likely to die in hospital (63% vs 47%), and people with a listed spouses were more likely to die in other settings (53% vs 37%; *p* = 0.007).^65^

### Illness factors

Eight studies examined illness factors associated with where people die, such as severity, type of disease and comorbidities.

#### Rural and urban studies

The only study to examine illness factors in rural and urban areas^47^ indicated that cancer type may have an impact on place of death, in addition to geographical location. Compared to urban areas, people with lung, brain, colorectal and prostate cancer in rural areas had a greater likelihood of dying at home.^47^

#### Urban studies

Six studies examined illness factors in urban areas, mostly around primary diagnosis. Three small studies in cancer populations found no significant effect of primary diagnosis, including cancer type, on place of death.^56,60,62^ Further, one study found that compared to people with dementia without a cancer co-morbidity, those with a primary diagnosis of cancer were less likely to achieve their preferred place of death (OR 0.5, 95% CI 0.28–0.97, *p* = 0.04).^63^

For people with dementia, a small study found dementia severity to have an impact on place of death. A larger percentage of people with severe dementia or cognitive impairment died in care homes and a smaller proportion in hospital, compared to people with mild dementia or no cognitive impairment.^53^ However, a higher proportion of those with no dementia/cognitive impairment died in a place that was not their usual address (79% vs 35%).

#### Coastal urban studies

For people with dementia, comorbidities have an impact of place of death. One study found a significantly greater proportion of people with diseases of the respiratory system died in hospital than in other places (*p* = 0.003).^65^

#### Environmental factors

Twelve studies examined environmental factors including, access to hospice or specialist palliative care, other supportive healthcare, and living situation.

#### Rural and urban studies

Two studies included cancer populations only. One study found that significantly more people in large metropolitan areas accessed hospice services than those in rural areas^45^ and of people who used hospice services, people in large metropolitan areas were less likely to die at home.^45^ In addition, home based hospice services were found to increase home deaths, particularly for people with cancer compared to dementia and this was more pronounced in rural areas (exp (ß6) = 1.3, 95% CI, 1.1–1.56, *p* = 0.001).^52^ Place of death was not affected by referral time to palliative care for people with cancer.^44^

#### Urban studies

Receipt of palliative care may influence where people with cancer died in urban areas. Compared to studies of populations who did not access palliative care, fewer hospital deaths were reported (24%–34% vs 50%–54%)^54,56,62^ and an increase in home (32% and 56%)^54,62^ and hospice deaths (39%)^56^ were reported. Late referral, or no referral at all to palliative care was more common for those dying in hospital, compared to home.^54^ Further, for people with cancer, where care was received facilitated their place of death. For example, those who spent less time in hospital and more time at home with support more often died at home.^24,60^ Specifically, home deaths were facilitated by increased contact with the GP, support from a cancer nurse specialist (such as Marie Curie)^24^ and visits from a specialised home care team.^60^ Further, home deaths were facilitated when relatives knew about incurability more than a week before death and were able to take more time off work^24^

One study identified contact with a healthcare professional as a facilitator to stating and, in turn, achieving preferred place of death for people with dementia^63^

#### Coastal urban studies

As identified in urban cancer populations, hospital care was associated with dying in hospital (OR 7.0, 95% CI 3.7–13.1), *p* < 0.001) in a coastal urban area too.^64^ However, advanced home care supported people to die at home.

For people with dementia living situation was associated with place of death. Those living at home alone or with family were significantly more likely to die in hospital, and those living in residential care more often remained there to die (*p* = 0.002).^65^

### Service factors

#### Urban studies

Only one study explored service factors associated with place of death. Madden et al.^59^ identified hospices in primary care trusts in London, UK and explored the association with hospice deaths for cancer patients. They found that four of seven of areas with the highest hospice deaths had a hospice in the area, and only one of the eight areas with the lowest hospice deaths had a hospice in the area. However, this was not a statistically significant.

## Discussion

This review is the first to examine the variation in place of death by rural, coastal and urban locations for people with dementia as compared to people with cancer. The evidence suggests that living in an urban area increases the likelihood of a person dying in hospital. This is aligned with existent evidence that includes deaths from all conditions.^2^ This is potentially due to the proximity of hospital to a person’s usual place of care, with more rural residents dying at home due to transport challenges hindering their ability to access healthcare.^33^ Further, some studies identified a dose-response relationship, with home, or care home, deaths becoming more likely the less urbanised the area got^40,41^ and hospital deaths more likely with increasing urbanisation.^51^ However, this message is less consistent amongst the included studies of cancer populations and varies across countries. It is likely that the results differ by country due to variation in public health policies and/or the commissioning or availability of rural health services.^50^ Factors were identified in the included studies that influenced these findings. Older age was found to decrease chances of dying at home for all rural residents,^66^ but the opposite for people in urban areas.^54,62^ Further, for urban residents the ability to communicate preferences enabled people to die where they wished to.^24,60,62,63^ It is likely that access to health and social care professionals and family or friends allows for communication of preferences, and in turn, allow for wishes to be fulfilled, and this is more likely for those with cancer than dementia.^28,67^ However, none of the associated factors were examined in all geographical locations, limiting comparisons.

In this review care home deaths were common for people with dementia, this may be due to the large proportion of studies being from the UK, reducing generalisability to all countries. In the UK, USA, Europe and other countries, people with dementia often move into care home in the last 1–2 years of life for 24-h care.^29,41,68–70^ In addition to the extended stay, ability to surround themselves with their own belongings and access to loved ones leads to residents feeling the care home is a substitute home environment for them when being at home may no longer be safe. This review has found that care home residents are less likely to be transferred to hospital at the end of life,^71^ may wish remain at the care home to die^63^ and can be supported to do so. Therefore, it may appropriate for future research to consider care homes as comparable with home as place of death for people with dementia.

This review has identified multiple gaps in the understanding of geographical variation in place of death. Firstly, this review identified very few studies examining place of death in rural and coastal areas. Just one study^66^ was identified that quantitatively examined place of death for people in rural areas alone and the associated factors. Much existent work focuses on the care received towards the end of life for people in rural areas.^13,33^ This work routinely cites travel and transport issues as primary barriers to accessing health and social care services for this population, which may have an impact on where people die. Further, the coastal studies that were included in this review^64,65^ did not consider the coastal context and the impact this may have on outcomes. In many countries, the older population living in rural and coastal areas has increased in recent years and is projected to continue to grow fastest in these areas^11,72–74^ making it a priority to understand where people are likely to die, and the associated factors. Careful consideration needs to be given to the coastal and rural contexts where services (including specialist services) may not exist, or where they do exist do not meet the demands of the area or population or are not of the appropriate quality. Understanding the dynamics that are unique to rural and coastal areas is important to ensure equitable service planning and delivery.^75^ This also includes consideration of cultural differences that exist between rural and urban residents, with rural residents often having strong cultural ties, through the environment or language, that are linked to a sense of self^76^ and may need to be maintained to support a ‘good’ death.^77^ This may mean that services or interventions developed in urban contexts may need to be adapted for the rural context.

The second gap identified within this review was the lack of a definition for coastal areas. The differences in health outcomes for coastal populations are emerging^11,78^ and work to develop a coastal definition is in it’s infancy.^79,80^ With the forementioned predicted growth of the older population and the increasing need for further research into these populations, a standardised definition of a coastal population would support identification and classification of population potentially at risk of poor health outcomes.^11,78^ Finally, this review particularly highlights the paucity of research on where people with dementia die and the associated factors while considering the rural, coastal or urban location. This review identified twice as many cancer studies to dementia. This could be a consequence of the limited advancement in dementia research, as compared to cancer, to date.

Ultimately, future research should seek to understand where coastal and rural populations die, particularly those with dementia, and where they may prefer to die to support efficient and equitable service planning and delivery for the growing population. This should include further examination of factors associated with place of death, with a particular focus on service factors, such as how service availability and accessibility impact on place of death and preferred place of death. Existent evidence suggests that place of death is associated with distance to services, such as hospice and hospital,^6,81^ but if and how this varies by condition remains unknown. Further, this review identifies a lack of understanding of how preferences may differ in urban, rural and coastal areas and across conditions. Geographical location should be considered more thoroughly in research into health outcomes.

### What this study adds

For the first time, this review examines the evidence on the impact geographical location has on place of death and how associated factors may vary by rural, coastal and urban locations. In particular, it focuses on populations who tend to receive access to care at the end of life (cancer), and for those who experience the most difficulties in accessing care (dementia), to identify specific groups that may be particularly affected by a rural, coastal and urban location. Future directions for research are proposed based on findings and gaps in the evidence base and the positioning of geographical location as a primary contributor of place of death is advocated for (Box 1).

**Box 1.** Implications for future research, policy and practice.

1. Coastal areas need to be defined and classified in the same way rural and urban locations are. 2. There is a need for increased recognition of the impact a rural, coastal and urban location may have on place of death. 3. Implications of living rurally for people with dementia need to be more widely considered. 4. Future research and service planning needs to ensure consideration of preferences and service factors, including availability and access, when examining place of death and how this may vary by rural/urban and coastal locations.

### Limitations

This review has limitations. There was large heterogeneity in the outcomes identified in the review which prevented the ability to undertake a meta-analysis and resulted in a largely descriptive review. In previous reviews, meta-analysis indicates factors such as age, marital status, and race have an association with place of death for people with dementia^34^ and function, preferences and home support^22^ for people with cancer. These factors have been identified within this review, but to a lesser extent and without the body of evidence to support a meta-analysis.

The inclusion criteria for this review may have limited the findings. For example, this review only included quantitative studies to enable direct comparisons across place of death. However, this will likely have precluded some contextual, in depth, understanding around preferences and quality of care found in qualitative studies. Due to the inclusion only of quantitative studies and most included studies were observational in design, the results are therefore limited to associations and causation cannot be established. Further, the parameters around the proportion of the sample population with the relevant conditions or age may have excluded some potentially important studies. For example, one study was excluded as it fell just under the threshold of 80% cancer population. However, inclusion of this study would not have significantly changed the results of this review as it found that most people with cancer in urban and coastal urban areas died in hospital.^82^ One interesting finding from this study was that more people in the urban coastal area wished to die in a care home, compared to the urban location who preferred to die at home. The authors suggest this might be due to the younger age of the urban sample.

It is also important to note that this review is potentially impacted by the underreporting of dementia on death certificates^83–85^ and its subsequent influence on research into place of death that relies heavily on death certificate data. This may have resulted in small sample sizes in the dementia studies included and the findings are cognisant of this.

Finally, we did not publish a protocol for this review. While this is important for reviews of clinical outcomes such as treatment effect estimations, this review was more exploratory of nature. Nevertheless, this is a limitation of the review.

## Conclusions

The evidence suggests that urban residents are more likely to die in hospital and rural residents are more likely to die at home, or in a care home. For all rural residents, increasing age reduced likelihood of dying at home and for all urban residents, indicating a preference for home increased likelihood of a home death. However, the evidence was stronger for those with cancer than dementia. More research is required that puts location in the forefront as a primary contributor to place of death and more consideration of the factors associated with place of death and how they vary by rural, coastal and urban locations is required.

## Supplemental Material

sj-docx-1-pmj-10.1177_02692163261426428 – Supplemental material for Unequal ends: A systematic review comparing place of death in rural, coastal and urban areas for older people with dementia to cancer Supplemental material, sj-docx-1-pmj-10.1177_02692163261426428 for Unequal ends: A systematic review comparing place of death in rural, coastal and urban areas for older people with dementia to cancer by India Tunnard-Johnson, Rachel L. Chambers, Peter May, Frank Jackson-Hill and Irene J. Higginson in Palliative Medicine
