# Supplementary material for: Unequal ends: A systematic review comparing place of death in rural, coastal and urban areas for older people with dementia to cancer
Source: Palliat Med. 2026 Mar 18;40(6):744–56. doi: 10.1177/02692163261426428 (PMC13221579; doi:10.1177/02692163261426428)
Supplement: sj-docx-1-pmj-10.1177_02692163261426428 – Supplemental material for Unequal ends: A systematic review comparing place of death in rural, coastal and urban areas for older people with dementia to cancer [file sj-docx-1-pmj-10.1177_02692163261426428.docx]

## Appendix 1. PRISMA checklist

| **Section and Topic** | **Item #** | **Checklist item** | **Location where item is reported** |
| --- | --- | --- | --- |
| **TITLE** | | |  |
| Title | 1 | Identify the report as a systematic review. | Page 1 |
| **ABSTRACT** | | |  |
| Abstract | 2 | See the PRISMA 2020 for Abstracts checklist. | Page 1-2 (some checklist items not applicable) |
| **INTRODUCTION** | | |  |
| Rationale | 3 | Describe the rationale for the review in the context of existing knowledge. | Pages 4-5 |
| Objectives | 4 | Provide an explicit statement of the objective(s) or question(s) the review addresses. | Page 5 |
| **METHODS** | | |  |
| Eligibility criteria | 5 | Specify the inclusion and exclusion criteria for the review and how studies were grouped for the syntheses. | Pages 6-8, and Table 1 |
| Information sources | 6 | Specify all databases, registers, websites, organisations, reference lists and other sources searched or consulted to identify studies. Specify the date when each source was last searched or consulted. | Page 6 |
| Search strategy | 7 | Present the full search strategies for all databases, registers and websites, including any filters and limits used. | Appendix 2 |
| Selection process | 8 | Specify the methods used to decide whether a study met the inclusion criteria of the review, including how many reviewers screened each record and each report retrieved, whether they worked independently, and if applicable, details of automation tools used in the process. | Pages 6-8 |
| Data collection process | 9 | Specify the methods used to collect data from reports, including how many reviewers collected data from each report, whether they worked independently, any processes for obtaining or confirming data from study investigators, and if applicable, details of automation tools used in the process. | Page 8 |
| Data items | 10a | List and define all outcomes for which data were sought. Specify whether all results that were compatible with each outcome domain in each study were sought (e.g. for all measures, time points, analyses), and if not, the methods used to decide which results to collect. | Page 8 |
|  | 10b | List and define all other variables for which data were sought (e.g. participant and intervention characteristics, funding sources). Describe any assumptions made about any missing or unclear information. | Page 8 |
| Study risk of bias assessment | 11 | Specify the methods used to assess risk of bias in the included studies, including details of the tool(s) used, how many reviewers assessed each study and whether they worked independently, and if applicable, details of automation tools used in the process. | Page 7 |
| Effect measures | 12 | Specify for each outcome the effect measure(s) (e.g. risk ratio, mean difference) used in the synthesis or presentation of results. | N/A |
| Synthesis methods | 13a | Describe the processes used to decide which studies were eligible for each synthesis (e.g. tabulating the study intervention characteristics and comparing against the planned groups for each synthesis (item #5)). | N/A |
|  | 13b | Describe any methods required to prepare the data for presentation or synthesis, such as handling of missing summary statistics, or data conversions. | N/A |
|  | 13c | Describe any methods used to tabulate or visually display results of individual studies and syntheses. | N/A |
|  | 13d | Describe any methods used to synthesize results and provide a rationale for the choice(s). If meta-analysis was performed, describe the model(s), method(s) to identify the presence and extent of statistical heterogeneity, and software package(s) used. | N/A |
|  | 13e | Describe any methods used to explore possible causes of heterogeneity among study results (e.g. subgroup analysis, meta-regression). | N/A |
|  | 13f | Describe any sensitivity analyses conducted to assess robustness of the synthesized results. | N/A |
| Reporting bias assessment | 14 | Describe any methods used to assess risk of bias due to missing results in a synthesis (arising from reporting biases). | N/A |
| Certainty assessment | 15 | Describe any methods used to assess certainty (or confidence) in the body of evidence for an outcome. | Page 7 |
| **RESULTS** | | |  |
| Study selection | 16a | Describe the results of the search and selection process, from the number of records identified in the search to the number of studies included in the review, ideally using a flow diagram. | Page 9 |
|  | 16b | Cite studies that might appear to meet the inclusion criteria, but which were excluded, and explain why they were excluded. | Page 18 |
| Study characteristics | 17 | Cite each included study and present its characteristics. | Table 2 |
| Risk of bias in studies | 18 | Present assessments of risk of bias for each included study. | Table 2 |
| Results of individual studies | 19 | For all outcomes, present, for each study: (a) summary statistics for each group (where appropriate) and (b) an effect estimate and its precision (e.g. confidence/credible interval), ideally using structured tables or plots. | Table 2 |
| Results of syntheses | 20a | For each synthesis, briefly summarise the characteristics and risk of bias among contributing studies. | Pages 9-17 |
|  | 20b | Present results of all statistical syntheses conducted. If meta-analysis was done, present for each the summary estimate and its precision (e.g. confidence/credible interval) and measures of statistical heterogeneity. If comparing groups, describe the direction of the effect. | N/A |
|  | 20c | Present results of all investigations of possible causes of heterogeneity among study results. | N/A |
|  | 20d | Present results of all sensitivity analyses conducted to assess the robustness of the synthesized results. | N/A |
| Reporting biases | 21 | Present assessments of risk of bias due to missing results (arising from reporting biases) for each synthesis assessed. | N/A |
| Certainty of evidence | 22 | Present assessments of certainty (or confidence) in the body of evidence for each outcome assessed. | N/A |
| **DISCUSSION** | | |  |
| Discussion | 23a | Provide a general interpretation of the results in the context of other evidence. | Pages 18-21 |
|  | 23b | Discuss any limitations of the evidence included in the review. | Page 20-21 |
|  | 23c | Discuss any limitations of the review processes used. | Page 20-21 |
|  | 23d | Discuss implications of the results for practice, policy, and future research. | Key statements and page 20 |
| **OTHER INFORMATION** | | |  |
| Registration and protocol | 24a | Provide registration information for the review, including register name and registration number, or state that the review was not registered. | Page 6 |
|  | 24b | Indicate where the review protocol can be accessed, or state that a protocol was not prepared. | Page 6 |
|  | 24c | Describe and explain any amendments to information provided at registration or in the protocol. | N/A |
| Support | 25 | Describe sources of financial or non-financial support for the review, and the role of the funders or sponsors in the review. | Page 22 |
| Competing interests | 26 | Declare any competing interests of review authors. | Page 22 |
| Availability of data, code and other materials | 27 | Report which of the following are publicly available and where they can be found: template data collection forms; data extracted from included studies; data used for all analyses; analytic code; any other materials used in the review. | Page 22 |

*From:*  Page MJ, McKenzie JE, Bossuyt PM, Boutron I, Hoffmann TC, Mulrow CD, et al. The PRISMA 2020 statement: an updated guideline for reporting systematic reviews. BMJ 2021;372:n71. doi: 10.1136/bmj.n71. This work is licensed under CC BY 4.0. To view a copy of this license, visit <https://creativecommons.org/licenses/by/4.0/>

## Appendix 2. Search strategy for all data bases

**MEDLINE**

|  | **Terms relating to place of death** | **Terms related to dementia and cancer** | **Terms related to geographical location** |
| --- | --- | --- | --- |
| MESH terms | Exp Hospitals/  Exp Hospices/  Exp Long-Term Care/  Exp Homes for the aged/  exp Terminal Care/ | exp dementia/ or exp Neoplasms/ | exp Hospitals, Rural/  exp Rural Health/  exp Rural Health Services/  exp Rural Nursing/  exp Rural Population/  exp Hospitals, Urban/  exp Urban Health/  exp Urban Population/ |
| Key terms | ((Place* or location* or site*) adj3 (Death* or dying or die*)).tw.  (palliative or hospice* or hospital* or home*).mp. | Dement*.tw.  Alzheimer*.tw.  (lewy* adj2 body).tw.  cancer*.tw.  neoplasm*.tw.  tumo*.tw.  malign*.tw.  carcino*.tw. | Rural*.mp.  Remote*.mp.  Coastal*.mp.  Seaside.mp.  Urban.mp.  City.mp.  Cities.mp.  Metropol*.mp. |
|  | OR | OR | OR |
|  | AND | AND | AND |

**PsycINFO**

|  | **Terms relating to place of death** | **Terms related to dementia and cancer** | **Terms related to geographical location** |
| --- | --- | --- | --- |
| MESH terms | exp Palliative Care/ or exp Terminally Ill Patients/  exp Hospitals/  exp Hospice/  exp Long Term Care/  exp Nursing Homes/  exp Residential Care Institutions/ | exp dementia/ or exp Neoplasms/ | exp Rural Health/  exp Rural environments/  exp Urban environments/  exp Urban Health/ |
| Key terms | ((Place* or location* or site*) adj3 (Death* or dying or die*)).tw.  (palliative or hospice* or hospital* or home*).mp. | Dement*.tw.  Alzheimer*.tw.  (lewy* adj2 body).tw.  cancer*.tw.  neoplasm*.tw.  tumo*.tw.  malign*.tw.  carcino*.tw. | Rural*.mp.  Remote*.mp.  Coastal*.mp.  Seaside.mp.  Urban.mp.  City.mp.  Cities.mp.  Metropol*.mp. |
|  | OR | OR | OR |
|  | AND | AND | AND |
|  | Limits: Human and English language | | |

**CINAHL**

|  | **Terms relating to place of death** | **Terms related to dementia and cancer** | **Terms related to geographical location** |
| --- | --- | --- | --- |
| Headings | (MH "Place of Death")  (MH "Hospitals+")  (MH "Hospices")  (MH "Long Term Care")  (MH "Nursing Homes+")  (MH "Residential Care+") | (MH "Dementia+")  (MH "Neoplasms+") | (MH "Hospitals, Rural") OR (MH "Rural Nurses") OR (MH "Rural Population") OR (MH "Rural Nursing") OR (MH "Rural Areas") OR (MH "Rural Health") OR (MH "Rural Health Services") OR (MH "Rural Health Centers")  (MH "Urban Areas") OR (MH "Urban Population") OR (MH "Hospitals, Urban") OR (MH "Urban Health") OR (MH "Urban Health Services") |
| Title and abstract | TI place* OR TI location* OR TI site*  AB place* OR AB location* OR AB site*  TI death* OR TI dyin* OR TI die*  AB death* OR AB dyin* OR AB die*  TX palliative OR TX hospice* OR TX hospital* OR TX home* | TI dementia* OR AB dementia*  TI Alzheimer* OR Alzheimer*  TI lewy body* OR AB lewy body*  TI cancer* OR AB cancer*  TI neoplasm* OR AB neoplasm*  TI tumo* OR AB tumo*  TI malign* OR AB malign*  TI carcino* OR AB carcino* | TX Rural* OR TX remote*  TX coastal OR TX seaside  TX coastal* OR TX seaside  TX urban OR TX city OR TX cities OR TX metropol* |
|  | OR | OR | OR |
|  | AND | AND | AND |

Embase

|  | **Terms relating to place of death** | **Terms related to dementia and cancer** | **Terms related to geographical location** |
| --- | --- | --- | --- |
| MESH terms | Exp Hospitals/  Exp Hospices/  Exp Long-Term Care/  Exp Homes for the aged/  exp "place of death"/ | exp dementia/ or exp Neoplasms/ | exp Hospitals, Rural/  exp Rural Health/  exp Rural Health Services/  exp Rural Nursing/  exp Rural Population/  exp Hospitals, Urban/  exp Urban Health/  exp Urban Population/ |
| Key terms | ((Place* or location* or site*) adj3 (Death* or dying or die*)).tw.  (palliative or hospice* or hospital* or home*).mp. | Dement*.tw.  Alzheimer*.tw.  (lewy* adj2 body).tw.  cancer*.tw.  neoplasm*.tw.  tumo*.tw.  malign*.tw.  carcino*.tw. | Rural*.mp.  Remote*.mp.  Coastal*.mp.  Seaside.mp.  Urban.mp.  City.mp.  Cities.mp.  Metropol*.mp. |
|  | OR | OR | OR |
|  | AND | AND | AND |
|  | Limit: Human | | |

**ASSIA**

((mainsubject.Exact("dementia" OR "cancer") OR summary(dementia*) OR summary(alzheimer*) OR summary(lewy* NEAR/4 /2 bod*) OR summary(cancer*) OR summary(neoplasm*) OR summary(tumo*) OR summary(malign*) OR summary(carcin*)) AND (mainsubject.Exact("place of death" OR "terminal care") OR summary(place* OR location* OR site*) AND (death* OR dyin* OR die*)) AND (mainsubject.Exact("remote areas" OR "hospitals" OR "rural communities" OR "rural population" OR "residential care" OR "rural populations" OR "rural-urban differences" OR "hospices" OR "urban areas" OR "urban health care" OR "coastal areas" OR "rural urban differences" OR "long term care" OR "rural health care" OR "urban health" OR "rural areas" OR "nursing homes") OR (summary(palliative) OR summary(hospice*) OR summary(hospital*) OR summary(home*) OR summary(rural*) OR summary(remote*) OR summary(coastal*) OR summary(seaside) OR summary(urban*) OR summary(city) OR summary(citi*) OR summary(metropol*)))) AND (stype.exact("Scholarly Journals") AND la.exact("ENG"))

## Appendix 3. Table of characteristics of included studies

| **Author, year, country** | **Aim** | **Design** | **Sample** | **Named Geographical location and geographical perspective** | **Place of death** | **Factors associated with place of death** | | | | **QualSyst score** |
| --- | --- | --- | --- | --- | --- | --- | --- | --- | --- | --- |
|  |  |  |  |  |  | **Individual factors** | **Illness factors** | **Environmental factors** | **Service factors** |  |
| **Rural vs urban studies (n=14)** | | | | | | | | | | |
| Bainbridge, 2015, Canada (39) | To determine factors associated with residents: having an emergency department visit in the last 6 months of life; and dying in acute care, that is, a hospital or an emergency department. | Population-based retrospective cohort study | Cancer N=1,196 | Ontario, Canada | No significant difference in odds of dying in hospital between rural and urban populations (OR 0.887, 95% CI 0.561-1.404, p=0.609) | NR | NR | NR | NR | 1 |
| Cohen, 2015, Belgium (40) | To describe the place of death of cancer patients and associated characteristics in 14 countries | Retrospective cohort study | Cancer  N=5,570,065 | France, Italy, England, Wales, Canada, Korea, Belgium, Netherlands, Spain, Mexico, Czech Republic, Hungary, New Zeland, US | A lower degree of urbanisation was related to higher chances of a cancer death at home, except in Canada and France where the opposite was true  Compared to strong urbanisation:  France - Average: OR 0.81, 95% CI 0.79-0.84; Rural: OR 0.91, 95% CI 0.88-0.95  Italy - Average: OR 1.19, 95% CI 1.15-1.22; Rural: OR 1.35, 95% CI 1.32-1.39  Spain - Average: OR 1.89, 95% CI 1.74-2.05; Rural: OR 1.78, 95% CI 1.52-2.08  Belgium - Average: OR 1.40, 95% CI 1.29-1.52; Rural: OR 1.47, 95% CI 1.32-1.63  Netherlands -Average: OR 1.18, 95% CI 1.11-1.24; Rural: OR 1.50, 95% CI 1.44-1.57  England - Average: OR 1.24, 95% CI 1.20-1.28; Rural: OR 1.41, 95% CI 1.28-1.55  Canada - Rural: OR 0.93, 95% CI 0.87-0.99  Korea - Average: OR 0.87, 95% CI 0.79-0.95; Rural: OR 1.24, 95% CI 1.16-1.33 | NR | NR | NR | NR | 1 |
| Cross, 2020, USA (41) | To assess trends and describe individual and geographic factors associated with place of death among ADRD patients in the United States | Cross-sectional study | Dementia  N=2,778,592 | USA | The largest proportion of nursing home deaths (65.2%) and the fewest hospice deaths (2.1%) occurred in nonmetro areas. The proportion of home deaths decreased as the areas became less urban: large metro 19.7%, medium metro 18.3%, small metro 17.6%, nonmetro 15.8%. | NR | NR | NR | NR | 1 |
| Fukui, 2011a (42) & 2011b (43), Japan | To identify predictors and determinants of home death of cancer patients receiving home palliative care | Cross-sectional survey | Cancer N=568 | Japan | 78 (13.7% of all) deaths occurred in a rural area. 40 at home, 38 in hospital. This finding was not significant or included in a regression model. | NR | NR | NR | NR | 0.8 |
| Howat, 2007, Australia (44) | To assess whether palliative care patients share factors which have predicted place of death in other, mainly urban, populations | Retrospective cohort study | Cancer N=270 | North Queensland, Australia | There was no statistically significant relationship between home death and geographical area.  There were significantly more patients from rural and remote areas where place of death was unknown (X2=26.6; df=1, p<0.001).  Home (n=54; 19%)  2 Metropolitan 49 (91%)  3-5 Rural 5 (9%)  Hospital (n=173; 64%)  2 Metropolitan 141 (82%)  3-5 Rural 24 (14%)  6+7 Remote 6 (3%)  Nursing home (n=17; 6%)  2 Metropolitan 14 (82%)  3-5 Rural 2 (11%)  6+7 Remote 1 (6%)  Unknown PoD (n=26; 10%)  2 Metropolitan 5 (19%)  3-5 Rural 11 (42%)  6+7 Remote 10 (38%) | NR | NR | All participants had palliative care support. Time of palliative care referral did not significantly vary by place of death. | NR | 0.91 |
| Jarosek, 2016, USA (45) | To use place-of-service (POS) codes in the Medicare hospice claims files to document where elderly hospice users with cancer die. | Retrospective cohort study | Cancer N=46,037 | USA | Larger proportion of urban residents dying at home and in hospice compared to rural.  Place of death for hospice users by geographical location N=30,629  Large metropolitan  Home 70.5%  Nursing home 13%  Hospital 7.2%  Hospice 8.5%  Other 0.9%  Metropolitan  Home 70.2%  Nursing home 12.6%  Hospital 3.9%  Hospice 12.2%  Other 1.1%  Urban  Home 73.4%  Nursing home 13.8%  Hospital 6.7%  Hospice 5.3%  Other 0.8%  Less urban  Home 66.5%  Nursing home 17%  Hospital 8.8%  Hospice - (numbers too small to provide)  Other -  Rural  Home 62.2%  Nursing home 19.9%  Hospital 11.1%  Hospice -  Other -  Chi square - p<.001 | NR | NR | Hospice users in the most urban areas were less likely to die at home (OR 0.61, 95% CI 0.48–0.77). Higher proportion of urban residents use hospice services.  Hospice users (n=30,629)  Large metropolitan 66.7%  Metropolitan 68.8%  Urban 62.3%  Less urban 61.1%  Rural 60.7%  Chi square - p<.001  Death at home of hospice users:  Large metropolitan 0.61 (0.48–0.77)  Metropolitan 0.83 (0.66–1.05)  Urban 1.25 (0.97–1.60)  Less urban 1.06 (0.84–1.34)  Rural ref  p<.001 | NR | 0.86 |
| Li, 2020, China (46) | To (1) examine the effect of socioeconomic status and healthcare utilization on place of death and (2) explore the mediating effect of healthcare utilization between socioeconomic status and place of death. | A population-based, retrospective study | Cancer N=894 | Yichang, China | Being on a rural insurance increased odds of dying in hospital (OR = 7.06, P < 0.001).  Hospital deaths (N=558) n(%)  UEBMI 24 (4.3)  URBMI 107 (19.17)  NRCMS 427 (76.52)  Home deaths (N=336) n(%)  UEBMI 157 (46.72)  URBMI 88 (26.19)  NRCMS 91 (27.08)  Chi(2)/U - 279.64, P<0.001 | For urban residents, being from a lower socioeconomic status (indicated by insurance type) increased odds of dying in hospital (OR =28.08, P < 0.001) | NR | NR | NR | 1 |
| Nilsson, 2020, Sweden (47) | To investigate if residency (rural versus urban) for cancer patients that have received palliative care and are included in the Swedish Register of Palliative Care (SPRC) is associated with a patient’s likelihood of dying at home compared to dying in hospital settings. | Retrospective cohort study | Cancer N=8,990 | Sweden | When adjusting for sex and age, all people in rural areas with cancer were more likely to die at home (data not provided). | NR | Patients with lung, brain, colorectal, and prostate cancer residing in rural municipalities all had a higher likelihood of dying at home than dying in a hospital, compared to those who lived in urban areas, holding age and sex constant.  Cancer type (n) - AOR (95% CI), p value (urban always ref)  Brain (n=561) - 1.54 (1.03-2.3) 0.035  Breast (n=621) - 1.31 (0.87-1.97) 0.19  Colorectal (n=1935) - 1.77 (1.43-2.18) <0.0001  Lung (n=5062) - 1.23 (1.06-1.43) 0.007  Prostrate (n=811) - 1.67 (1.22-2.29) 0.001 | NR | NR | 0.86 |
| Park, 2024, Canada (48) | To examine differences in the use of end-of-life acute care and the location of death among residents with dementia in rural long term care (LTC) homes, compared with those in urban LTC homes. | Retrospective cohort study | Dementia N=63,375 | Ontario, Canada | Compared to rural areas, people in urban areas were less likely to die in a LTC home. The proportion dying in hospital was also higher (30.4% vs 17.5%)  Adjusted (for sex, age, income quintile and no of conditions) Relative Risk to dying in a care home (all compared to rural areas) (unadjusted risks were very similar)  RR (95% CI)  Urban 1 - 0.84 (0.83-0.85)  Urban 2 - 1.01 (1.00-1.02)  Urban 3 - 0.99 (0.98-1.00)  Urban 4 - 1.00 (0.99-1.02) | NR | NR | NR | NR | 1 |
| Rashid, 2024, USA (49) | To evaluate the association of racial/ethnic factors with disparities in the location of death. | Cohort study | Cancer N=815,780 | USA | Increased proportion of home deaths in non-metropolitan areas and hospital and hospice deaths in metropolitan areas.  Metro n(%)(N=788,499)  Inpatient hospital 199,749 (25.3)  Home 430,899 (54.6)  Nursing home 65,303 (8.3)  Hospice 72,676 (9.2)  Outpatients/ED 19,872 (2.5)  Non-metro n(%) (N=27,281)  Inpatient hospital 2,461 (9)  Home 23,139 (84.8)  Nursing home 814 (3)  Hospice 619 (2.3)  Outpatients/ED 248 (0.9) | NR | NR | NR | NR | 0.95 |
| Raziee, 2017, Canada (50) | To determine factors associated with home death in patients with cancer in Ontario, particularly to assess the association between death at home and (1) patients’ rural/urban residence and (2) neighbourhood income in urban areas. | Retrospective cross-sectional study | Cancer N=193,783 | Ontario, Canada | When adjusted for other covariates, rural residence was associated with a lower likelihood of home death as compared to urban (OR: 0.828, 95% CI: 0.801-0.856). | Living in the highest income urban neighbourhood was associated with a 69% increase in likelihood of dying at home compared to the lowest (OR: 1.692, 95% confidence interval [CI]: 1.543-1.855).  Income quintile of urban areas: AOR (95% CI)  1 (lowest quintile) 1.000 1.000  2 1.198 (1.102-1.304)  3 1.289 (1.193-1.394)  4 1.378 (1.246-1.522)  5 (highest quintile) 1.692 (1.543-1.855) | NR | NR | NR | 0.95 |
| Reyiners, 2015, Belgium (51) | To examine variations in place of death of older people who died from a dementia-related disease and their association with sociodemographic factors, social support, residential, and healthcare system factors in European and non-European countries | Retrospective cohort study | Dementia N=264,604 | Belgium, Canada (Quebec excluded), Czech Republic, England, France, Hungary, Italy, Mexico, the Netherlands, New Zealand, Spain (Andalusia; 2010), South Korea, United States (2007), and Wales. | In England, France, Italy, Spain, Hungary, Canada, and South Korea, the likelihood of dying in a hospital setting was higher in stronger in urbanized regions. Findings not significant for The Netherlands, Belgium, and Wales.  Hospital death vs elsewhere OR (95% CI)  Urbanization level: very strong/strong (vs average/weak/rural)  England 1.5 (1.4-1.6)  France 1.1 (1.0-1.2)  Italy 1.3 (1.3-1.4)  Spain 1.3 (1.1-1.6)  Hungary 2.7 (2.2-3.3)  Canada 1.2 (1.1-1.3)  South Korea 1.6 (1.4-1.9) | NR | NR | NR | NR | 0.91 |
| Sleeman, 2014, UK (3) | To examine trends in place of death in dementia in England, and the individual and regional factors associated with place of death over a 10 year period. | Population-based cross-sectional study | Dementia N=397,513 | England | People in rural areas have a higher likelihood of dying at home or in a hospice, than those in urban areas.  Care home vs hospital PR (95% CI)  Urban 1.00 - -  Semi-rural 1.10 (1.08-1.12)  Rural 1.17 (1.15-1.19)  Home/hospice vs hospital PR (95% CI)  Urban 1.00 - -  Semi-rural 1.12 (1.07-1.17)  Rural 1.52 (1.46-1.59) | NR | NR | NR | NR | 1 |
| Yun, 2023, South Korea (52) | To explore the changes in the place of death of patients with cancer after the introduction of home-based hospice care in Korea | Retrospective cohort study | Dementia (n=11063) and cancer (n=130435)  N=218,522 | South Korea | NR | NR | NR | After receiving home based hospice services, people with cancer were more likely to die at home this increase was more than found for people with dementia (control group) and pronounced in rural areas (exp [ß6]= 1.320 [95%CI, 1.118-1.558]; P = .001). | NR | 0.95 |
| **Rural studies (n=1)** | | | | | | | | | | |
| Black, 2016, Scotland (67) | To examine predictors of place of death over an 11-year period between 2000 and 2010 in Dumfries and Galloway, south west Scotland. Primary aim was to determine whether death at home was becoming more or less common. Second, we wanted to explore the relation between age, gender, cause and place of death and the extent to which our hospital’s specialist palliative care unit (SPCU) impacted acute hospital deaths. | Retrospective cohort study | Dementia (n=545) and cancer (n=3105)  N=19,697 | Dumfries and Galloway, Scotland | **Dementia, n (%)**  Cottage hospital: 42 (8.5)  Home: 47 (9.5)  Residential care: 354 (71.7)  Acute Hospital: 51 (10.3)  SPCU: 3 (0.6)  Not SPCU: 48 (9.7)  **Cancer, n (%)**  Cottage hospital: 321 (15.7)  Home: 569 (27.8)  Residential care: 97 (4.7)  Acute Hospital: 1059 (51.8)  SPCU: 549 (26.8)  Not SPCU:510 (24.9) | People were less likely to die at home as they got older, irrespective of cause of death (OR 0.98, 95% CI 0.97-0.98). Older people with dementia were particularly unlikely to die in an acute hospital and very likely to die in a residential home (OR 20.26, CI 95% 14.51 - 28.29) | NR | NR | NR | 0.9 |
| **Urban studies (n=12)** | | | | | | | | | | |
| Fleming, 2017, UK (53) | To examine associations between factors potentially related to reported comfort during very old people’s final illness: physical and cognitive disability, place of care and transitions in their final illness, and place of death. | Prospective cohort study | Dementia N=180 | Cambridge, UK | N (%)  Home 19 (11)  Long-term care 76 (42)  Hospital 85 (47) | NR | Compared to no cognitive impairment or a mild dementia/ impairment, a higher proportion of people with severe dementia died in a care home (76% vs 21% and 23%) and a smaller proportion died in hospital (20% vs 67% and 62%).  No dementia/cognitive impairment (n=33)  N(%)  Home: 4 (12)  Long-term care: 7 (21)  Hospital: 22 (67)  Place of death not usual address Y - 26 (79)  Minimal-mild dementia/moderate cognitive impairment (n=81)  N(%)  Home: 12 (15)  Long-term care: 19 (23)  Hospital: 50 (62)  Place of death not usual address Y - 56 (69)  Moderate-severe dementia/severe cognitive impairment (n=66)  N(%)  Home: 3 (5)  Long-term care: 50 (76)  Hospital: 13 (20)  Place of death not usual address Y - 23 (35) | NR | NR | 0.85 |
| Gomes, 2015, UK (54) | To determine the association between place of death, health services used, and pain, feeling at peace, and grief intensity | Case control survey | Cancer N=881 | London, UK | Home death: n=175  Hospital death: n=177 | Home death was more likely when patients discussed their preferences with family (AOR, 3.39; 1.63–7.04). | NR | Home death odds were higher when relatives knew of incurability >1 week before death (AOR, 6.51; 2.84–14.91) and if relatives took >14 days off work in the last 3 months before the patient died (AOR, 9.94; 2.56–38.65 versus <4 days off) or were not working (AOR, 3.70; 1.28–10.68)  When patients stayed 15–28 days in hospital during the last 3 months of life, the odds of dying at home were 75% lower compared to patients with 0–7 days in hospital (adjusted odds ratio (AOR), 0.25; 0.10–0.61). Furthermore, the odds decreased by 91 % for patients who were in hospital for more than 28 days in the last 3 months of life (AOR, 0.09; 0.03–0.25)  Patients who received 3 or more GP home visits in their last 3 months of life had six times greater odds of dying at home compared to patients with just one or no GP home visits (AOR, 6.27; 2.83–13.91). Those with two visits also had greater odds of dying at home (AOR, 3.42; 1.23–9.55).  Only 4% of those who died in hospital had care from Marie Curie nurses, compared to 41% who died at home. | NR | 1 |
| Hales, 2014, Canada (55) | To explore the quality of dying and death of a sample of advanced cancer patients in an urban Canadian setting and examine the relationship of the quality of dying and death to length of specialized palliative care received and to place of death | Cross-sectional survey | Cancer N=402 | Toronto, Canada | 130 (32%) patients died at home, 152 (38%) in an inpatient hospice/ palliative care unit, 9 (2%) in a long-term care facility, and 111 (28%) in an acute care hospital setting. | People who died at home were significantly older than those who died in hosptial or in LTC (p=0.003) | Those who died at home or in LTC had sig longer time between diagnosis to death (p=0.003) | Receipt of late or no palliative care was most common among those dying in hospital followed by those dying in hospice/ palliative care unit/care facility and least common among those dying at home (p<0.001) | NR | 0.95 |
| Higginson, 2017, UK (57) | To determine the associates with the most and least preferred place of death, treatment priorities and whether these are similar to or different from the associates with actual place of death across three countries with developed specialist palliative care. | Prospective cohort study | Cancer  N=163  (70 in London, 70 in Dublin and 23 in New York. New York sample not included in analysis of factors associated with actual PoD, total n=79) | London, UK  New York, USA  Dublin, Ireland | N (%)(total N=103)  Home 22 (21)  Home of a friend/relative 0(0)  Palliative care unit or inpatient hospice 40 (39)  Hospital 35 (34)  Care home 6 (6)  Elsewhere 0 (0) | Age had no significant impact on place of death.  Participants who valued only quality of life (OR 3.11, 95% CI 2.89–3.36) were more likely to die at home than those who valued both quality and extension of life. | Primary diagnosis had no significant impact on place of death.  Higher functional independence (OR 1.05, 95% CI 1.04–1.06) was associated with greater likelihood of dying at home. | All participants received palliative care support. | NR | 1 |
| Houttekier, 2009, Belgium (56) | To examine determinants of place of death in the Brussels metropolitan region for patients who died after chronic illness, and thus possibly could have benefited from receiving palliative care. | Retrospective cohort study | Dementia (n=155) and cancer (n=2431)  N=3,672 | Brussels, Belgium | Of all cancer patients, 72.4% died in hospital, which is more than that in any other disease. Noncancer conditions were more likely to die at home (AOR 1.61; 95% CI 1.30-2.00) or care home (AOR 1.75, 95% CI 1.21- 2.53) than cancer conditions’=.  **Cancer**  % of deaths  Home 15.7  Hospital 72.4  Care home 11.6  Elsewhere 0.3  **Alzheimer's**  % of deaths  Home 9.0  Hospital 16.8  Care home 74.2  Elsewhere 0.0 | NR | NR | NR | NR | 0.82 |
| Koffman, 2014, UK (58) | To investigate if place of death for those who died in London from all cancer causes differed according to geographical origin (i.e. country of birth) and over time. | population-based retrospective cohort study | Cancer N=93,375 | London, UK | Place of death n, %  Hospice 16,972, 18.2%  Own residence 17,445, 18.7%  Hospital 49,032, 52.5%  Other communal 8,757, 9.4%  Elsewhere 1,169, 1.3% | Deaths in hospital settings were less likely among those born in Ireland (PR 0.92 [0.90–0.95]), but more likely for those born in Asia, including China (PR 1.12 [1.08–1.15]) and Africa (PR 1.11 [1.07–1.16]). Compared to all other settings, deaths in hospice settings were significantly less likely among those born in Asia, including China (PR 0.73 [0.68– 0.80]), Africa (PR 0.83 [0.74–0.93]) and ‘other’ locations (PR 0.90 [0.82–0.98]). Deaths in decedents’ own homes were significantly less likely among those born in the Caribbean (PR 0.91 [0.85– 0.98]) and more likely among those born in Ireland (PR 1.13 [1.07–1.19]), compared to all other locations. Finally, deaths in other communal establishments including nursing and residential care homes were more likely for those born in Ireland (PR 1.01 [1.01–1.19]) and less likely for those born in Europe (PR 0.80 [0.73–0.88]), Asia, including those from China (PR 0.64 [0.55– 0.74]) and Africa (PR 0.77 [0.62–0.95]). | NR | NR | NR | 0.91 |
| Livingston, 2013, UK (59) | To improve end-of-life care for people with dementia in a care home by increasing the number and implementation of advanced care wishes. | Before and after, non-randomised, intervention study | Dementia N=98 | London, UK | An intervention to improve end of life care discussions and ACP increased the number of people with dementia remaining in the care home to die 47% to 76%, χ2(1) = 5.3, p = 0.02 | NR | NR | NR | NR | 0.95 |
| Madden, 2011, UK (60) | (1) to explore cancer mortality data for deaths in home, hospice, hospital and nursing homes for 2002–2007 using maps and funnel plots; and (2) to compare year-on-year data to identify PCTs with consistently high or low performance or changes in place of death. | Retrospective cohort study | Cancer  2005: n=13,858  2006: n=13,770  2007: n=13,277 | London, UK | The map shows a tendency for higher proportions of home deaths in outer London primary care trusts (PCT).  Home n (% of all deaths in that year)  2005 2591 (18.7)  2006 2675 (19.4)  2007 2588 (19.5)  Hospice n (% of all deaths in that year)  2005 2646 (19.1)  2006 2768 (20.1)  2007 2687 (20.12)  Hospital n (% of all deaths in that year)  2005 7465 (53.9)  2006 7097 (51.5)  2007 6679 (50.3)  Nursing home n (% of all deaths in that year)  2005 1001 (7.2)  2006 1070 (7.8)  2007 1104 (8.3)  Other/unknown n (% of all deaths in that year)  2005 155 (1.1)  2006 160 (1.2)  2007 219 (1.6) | NR | NR | NR | Of the seven PCTs with the highest proportion of hospice deaths, four contained a hospice. Conversely, of the eight PCTs with the lowest propor tion of hospice deaths, only one contained a hospice, although this finding was not statistically significan (Fisher’s exact p-value = 0.12). | 0.8 |
| Nakamura, 2010, Japan (61) | To investigate factors affecting terminally ill cancer patients to die at home | Cohort study | Cancer N=92 | Nagoya, Japan | Home n=60  Hospital n= 32 | No significant differences in place of death by age (p=0.42).  In univariate analyses, there were significant differences in preferences for place of death and where people died. In multivariate analyses, patients with no preference regarding the place of death or a preference for home death were more likely to die at home (vs preference for hospital death, OR = 5.12, 95% CI = 1.37–19.14, OR = 16.53, 95% CI = 3.30–82.73, respectively). | No significant differences in place of death by type of cancer (p=0.71). | No differences in place of death for those living with or without family (p=0.09).  Those who had more visits from the family physician and nurses died at home more often (p=0.01 and p=0.03 respectively)  In multivariable analyses, patients of families with no preference regarding the place of death or a preference for home death were more likely to die at home (vs family preference for hospital death, OR = 8.04, 95% CI = 2.08–31.08, OR = 136.57, 95% CI = 18.84– 1347.51, respectively) | NR | 0.95 |
| Sampson, 2018, UK (62) | To 1. Describe the course of physical and psychological  symptoms; 2. Examine health and social care service utilisation; 3. Describe the care received at the end of life. | Prospective cohort study | Dementia  N=85 (n=32 died) | London, UK | Place of death n(%)  Nursing home 26 (81)  Own home 1(3)  Acute hospital 5 (16) | NR | NR | NR | NR | 0.85 |
| Varani, 2015, Italy (63) | To analyze demographic and clinical variables that influence the place of death in a sample of cancer patients who receive palliative home care | Retrospective observational study | Cancer N=1,374 | Bologna, Italy | NR | Older age was correlated with home deaths, compared to hospital (OR 0.98, 95%CI 0.966-0.996, p=0.013), but not hospice.  Awareness of diagnosis was not associated with place of death in the multivariable analysis (OR=1.404 95% CI 0.788–2.502, p=0.249) | No significant differences in place of death by type of cancer.  Origin of cancer n(%)  Head and neck  Total 31 (2.3)  Home death 12 (1.5)  Hospital death 13 (3.7)  Hospice death 6 (2.8)  Gastrointestinal  Total 493 (35.9)  Home death 305 (37.7)  Hospital death 116 (33)  Hospice death 72 (34)  Thoracic  Total 303 (22.1)  Home death 171 (21.1)  Hospital death 79 (9.8)  Hospice death 53 (25)  Breast  Total 102 (7.4)  Home death 59 (7.3)  Hospital death 26 (7.4)  Hospice death 17 (8)  Genitourinary  Total 250 (18.2)  Home death 152 (18.8)  Hospital death 62 (17.6)  Hospice death 36 (17)  Neurological  Total 47 (3.4)  Home death 30 (3.7)  Hospital death 9 (2.6)  Hospice death 8 (3.8)  Hematologic  Total 54 (3.9)  Home death 34 (4.2)  Hospital death 18 (5.1)  Hospice death 2 (1)  Other  Total 94 (6.8)  Home death 44 (5.4)  Hospital death 32 (9.1)  Hospice death 18 (8.5) | Those who received palliative home care, 810 (56%) died at home, 352 (24%) died in hospital, and 212 (15%) died in hospice.  Home visits from a physician was associated with home deaths, compared to hospital (OR=0.951, 95% CI 0.929-0.973, p=0.000) and hospice (OR=0.943, 95% CI 0.917-0.971, p=0.000). The number of hospital admissions was associated with deaths in hospital (OR=7.387, 95% CI 4.535-12.034, p=0.000) and hospice (OR=7.489, 95% CI 4.452-12.598, p=0.000). | NR | 0.86 |
| Wiggins, 2019, UK (64) | To understand preferences for place of death among people with dementia, and to identify the factors associated with achieving these preferences. | Retrospective cohort study | Dementia N=1,047 | London, UK | Actual place of death was documented for 974 (93.0%) people. Of those, 533 (50.9%) died in a care home, 277 (26.5%) died at home, 127 (12.1%) died in hospital and 33 (3.2%) died in a hospice. | Preferred place of death was achieved by 672 (83.7%) of the people for whom both the preferred and actual place of death were known and not achieved by 131 (16.3%). | A primary diagnosis of cancer was associated with decreased odds of achieving preferred place of death (OR 0.52 (95% CI 0.28–0.97) p=0.039) | All participants had contact with a healthcare professional to discuss preferences for care at the end of life. | NR | 0.95 |
| **Coastal urban (n=2)** | | | | | | | | | | |
| Ahlner-Elmqvist, 2004, Sweden(65) | To evaluate time home, to spent at home, place of death and differences in sociodemographic and medical characteristics of patients, with cancer in palliative stage, receiving either hospital-based advanced home (AHC), including 24-hour service by multidisciplinary palliative conventional hospital team a care or (CC). | Prospective non randomised study | Cancer N=280 | Malmo, Sweden | NR | NR | NR | Compared to conventional hospital care, a higher proportion of participants receiving advanced home care died at home (38.5% vs 6.1%) and fewer in hospital (18.8% vs 38.7%). Support received from the conventional hospital care was associated with dying in hospital (OR 7.0, 95% CI 3.7-13.1), p<0.001).  Irrespective of home care received, living alone (vs with someone else) was associated with death in hospital (OR 2.4, 95% CI 1.2-4.7, p=0.014). | NR | 0.82 |
| Triandafilidis, 2024, Australia | To investigate end-of-life care for people with dementia in a regional area of Australia, specifically (1) what are the characteristics of people who die with dementia in hospital, compared to other settings; (2) what is the end-of-life care provided to people who die with dementia in hospital, compared to other settings; and (3) if there is any association between declining function and death for people with dementia | Retrospective clinical audit | Dementia N=705 | Greater Sydney and coastal New South Wales, Australia | 346 (49.1%) in hospital, and 359 (50.9%) in other settings. A subset of 299 people were selected at random for further analysis. Of these, 177 people died in hospital, and 122 in other settings. | People without spouses listed were more likely to die in hospital (63% vs 47%), and people with spouses listed were more likely to die in other settings (53% vs 37%; P = 0.007) | Diseases of the respiratory system were significantly more likely to be the primary diagnosis for people dying in hospital (P = 0.003) | A significant difference was found in living situation, with people dying in hospital more likely to be living at home, either with family (41% vs 29%) or alone (15% vs 6%) and those dying in other settings more likely to be living in RACFs (64% vs 44%; P = 0.002) | NR | 0.91 |
